# Anti-inflammatory activity of cinnamon water extract *in vivo* and *in vitro* LPS-induced models

**DOI:** 10.1186/1472-6882-12-237

**Published:** 2012-11-28

**Authors:** Joung-Woo Hong, Ga-Eun Yang, Yoon Bum Kim, Seok Hyun Eom, Jae-Hwan Lew, Hee Kang

**Affiliations:** 1 Graduate School of East–west Medical Science, Kyung Hee University, Yongin, Gyeonggi, 446-701, South Korea; 2 College of Oriental Medicine, Kyung Hee University, Seoul, 130-701, South Korea; 3 College of Life Science, Kyung Hee University, Yongin, Gyeonggi, 446-701, South Korea

**Keywords:** Cinnamon, Anti-inflammatory activity, TNF-α, Signaling, Polyphenols

## Abstract

**Background:**

Cinnamon bark is one of the most popular herbal ingredients in traditional oriental medicine and possesses diverse pharmacological activities including anti-bacterial, anti-viral, and anti-cancer properties. The goal of this study is to investigate the *in vivo* and *in vitro* inhibitory effect of cinnamon water extract (CWE) on lipopolysaccharide (LPS)-induced tumor necrosis factor (TNF)-α and its underlying intracellular mechanisms.

**Methods:**

CWE was orally administrated to mice for 6 days prior to intraperitoneal injection of LPS. Serum levels of TNF-α and interleukin (IL)-6 were determined 1 hour after LPS stimulation. Peritoneal macrophages from thioglycollate-injected mice were isolated and assayed for viability, cytokine expression and signaling molecules upon LPS stimulation. CWE was further fractioned according to molecular size, and the levels of total polyphenols and biological activities of each fraction were measured.

**Results:**

The oral administration of CWE to mice significantly decreased the serum levels of TNF-α and IL-6. CWE treatment *in vitro* decreased the mRNA expression of TNF-α. CWE blocked the LPS-induced degradation of IκBα as well as the activation of JNK, p38 and ERK1/2. Furthermore, size-based fractionation of CWE showed that the observed inhibitory effect of CWE *in vitro* occurred in the fraction containing the highest level of total polyphenols.

**Conclusions:**

Treatment with CWE decreased LPS-induced TNF-α in serum. *In vitro* inhibition of TNF-α gene by CWE may occur via the modulation of IκBα degradation and JNK, p38, and ERK1/2 activation. Our results also indicate that the observed anti-inflammatory action of CWE may originate from the presence of polyphenols.

## Background

The bark of cinnamon has been used not only as a spice and tea, but also as one of the key components of herbal remedies for the common cold, cardiovascular disease, and chronic gastrointestinal and gynecological disorders in oriental herbal medicine. Accordingly, extensive studies on the pharmacological activities of the cinnamon bark have been conducted, indicating that cinnamon bark is involved in a vast range of pathological and physiological events. For instance, essential oil and water-based extracts from cinnamon have been shown to be effective against pathogenic microbes, viruses, and various types of tumor cell lines [1-4]. Furthermore, it has been also reported that cinnamon bark reduces the level of serum glucose through the enhancement of insulin-regulated glucose utilization *in vivo*[5,6].

Inflammation is a protective response for the purpose of removal of exogenous and endogenous harmful substances produced by injurious stimuli and is a part of the healing process in wounded tissues [7]. Since proinflammatory cytokines such as tumor necrosis factor-alpha(TNF-α), interleukin(IL)-1 and IL-6, lipid mediators, proteases, and oxidants produced during the typical response can cause damage to normal tissues regardless of how and where the inflammatory response is triggered, the substances involved in the inflammatory response need to be tightly regulated. If the scavenging reaction is delayed, the inflammatory response may evolve into a variety of chronic inflammatory diseases, such as atherosclerosis, rheumatoid arthritis, asthma, and neurodegenerative diseases. A vast number of molecular studies have identified several target molecules involved in inflammatory changes, and most anti-inflammatory drugs currently used suppress the biosynthesis of the inflammatory mediators mentioned earlier [8].

Previous studies have indicated that the major pharmacological activities of cinnamon bark, such as its anti-bacterial, anti-inflammatory, anti-viral, and anti-cancer effects are derived from essential oils such as cinnamaldehyde [1,9-11]. However, since cinnamon bark has been typically used as in the form of a water extract, where the volatile ingredients are seldom found, it is likely that the established pharmacological activities of cinnamon bark depend on a mixture comprised of a variety of water-soluble components, thereby ensuring its safety as a traditional remedy. Recently, it has been found that cinnamon bark water extract (CWE) elevates glucose uptake through the promotion of insulin sensitivity and inhibits angiogenesis through blocking vascular endothelial growth factor 2 signaling [12,13]. These results indicate that the observed pharmacological activities may have originated from polyphenolic compounds in CWE.

In this study, we investigated the *in vivo* and *in vitro* effects of CWE on lipopolysaccharide (LPS)-induced TNF-α and its underlying intracellular mechanisms. We also fractioned CWE according to molecular size to determine whether there exists a positive correlation between the anti-inflammatory activity of CWE and the amount of polyphenolic compounds.

## Methods

### Preparation of cinnamon water extract

Cinnamon bark (*Cinnamomi cassia* P_RESL_) of Vietnamese origin was purchased from Omni Herb (Daegu, South Korea). The plant was identified by Professor Choi of the Department of Herbology at Kyung Hee University. A voucher specimen sample (CC-2011) was deposited at the Laboratory of Herbology at Kyung Hee University. The plant was pulverized and soaked in one volume of water for 48 hours at room temperature, and further dissolved by sonication for 1 hour. The extract was filtered and evaporated using a freeze dryer (EYELA, Japan) at −70°C. The yield of CWE was about 3.62%. For size fractionation, 1.28 g of CWE was dissolved in 30 ml of distilled water and fractions were collected using 3 kDa and 10kDa Amicon Ultra Centrifugal Filter device (Millipore, Ireland). The yields of a low molecular weight (MW) fraction (below 3 kDa), a middle MW fraction (between 3 kDa and 10 kDa), and a high MW fraction (over 10 kDa) were 60%, 15% and 25% of CWE, respectively. All the final samples were dissolved in PBS and sterilized by passing through a 0.22-μm syringe filter.

### Animals

Eight-week-old male BALB/c mice were purchased from the Korean branch of Taconic, SamTaco (Osan, Korea) and fed rodent chow and water *ad libitum* in a temperature- and humidity-controlled pathogen-free animal facility at the Medical Center of Kyung Hee University Hospital. Mice were maintained in accordance with the Guide for the Care and Use of Laboratory Animals issued by the US National Research Council (1996), and the protocol KHMC-IACUC12-006 was approved by the Kyung Hee University Medical Center Institutional Animal Care and Use committee.

### *In vivo* LPS injection

CWE (20, 100 or 500 mg/kg of body weight) was given to mice via oral gavage for 6 days. Control mice received an equal volume of normal saline during the experimental period. Each group consisted of 12 mice. On day 7, LPS (serotype 055:B5; Sigma, St. Louis, MO, USA) (1.3 mg/kg) was injected intraperitoneally 1 hour before blood sampling. Blood was obtained by cardiac puncture. As a reference drug, dexamethasone (Sigma) (5 mg/kg) was injected intraperitoneally 18 hours before the LPS injection. Blood samples were centrifuged at 800 g for 20 min. The serum samples obtained were stored at −20°C until used.

### Isolation and culture of peritoneal macrophages

For the use of *in vitro* culture of macrophages, normal mice were injected intraperitoneally with 2 ml of sterile thioglycollate medium (BD, France), and macrophages were collected three days later by peritoneal gavage with cold Dulbecco’s modified Eagle’s medium (DMEM). The recovered peritoneal fluid was washed by centrifugation. The cells were resuspended in DMEM with 10% fetal bovine serum and incubated for 3 hours at 37°C with 5% CO_2_. Non-adherent cells were removed.

### Viability assay

Cell viability was determined using the MTT method. Macrophages were seeded in 96-well plates and treated with 10, 50, 100, 200, and 400 μg/ml CWE in the presence or absence of LPS for 24 hours. Ten microliters of MTT solution (5 mg/ml) (Sigma) was added to each well and, after 2 hours of incubation, media was aspirated and 100 μl of dimethyl sulfoxide (DMSO) (Sigma) was added. The optical density was read at 560 nm using a microplate reader (Molecular Devices, Sunnyvale, CA, USA).

### cDNA preparation and real-time PCR

Peritoneal macrophages were seeded in 6-well plates and pre-treated with CWE for 1 hour, then stimulated with 100 ng/ml LPS for 4 hours. Total RNA was isolated using an RNeasy Mini Kit (Qiagen, Germany) and cDNA was reverse-transcribed using Superscript III reverse transcriptase (Invitrogen, Carlsbad, CA, USA). Diluted cDNA was mixed with Power SYBR Green PCR Master mix (Applied Biosystems, Foster City, CA, USA) and 2 pmol of primers for TNF-α or GAPDH and. The following forward and reverse primer sequences were used: TNF-α, forward :5^′^- ATG ATC GCG GAC GTG GAA-3′ and reverse: 5^′^-AGG GCC TGG AGT TCT GGA A-3′; GAPDH, forward: 5^′^-GGC ATG GAC TGT GGT CAT GA-3^′^ and reverse: 5^′^-TTC ACC ACC ATG GAG AAG GC-3^′^. Amplification of cDNA was performed in triplicate using a StepOne realtime PCR system (Applied Biosystems). After an initial heat denaturation at 95°C for 10 min, the PCR conditions were set at 95°C for 15 s and 60°C for 1 min for 40 cycles. For each PCR, a corresponding mRNA sample without RT was included as a negative control. Quantification of each cDNA copy number was determined according to the manufacturer’s protocol. The GAPDH gene was used as an endogenous control.

### Cytokine measurement

The levels of cytokines from serum or cell supernatants were measured by enzyme-linked immunosorbent assay (ELISA), according to the manufacturer’s protocol (BD Pharmingen, USA).

### Western blot analysis

Peritoneal macrophages were seeded and pretreated with CWE for 1 hour and then stimulated with LPS for 15 min. Cells were rinsed in cold PBS and then lysed on ice in 0.1 ml of RIPA buffer (50 mM Tris–HCl, pH 7.5; 150 mM NaCl; 1 mM EDTA; 20 mM NaF; 0.5% NP-40; and 1% Triton X-100) containing phosphatase inhibitor cocktail (Sigma) and protease inhibitor cocktail (Roche Diagnostics, Mannheim, Germany). After centrifugation at 13,000 g for 10 min, supernatants were collected. Protein concentrations were determined using the Bradford protein assay reagent (Bio-Rad, USA) and the samples were diluted with 6x sodium dodecyl sulfate(SDS) buffer and boiled for 3 min. The samples were separated on a 10% SDS-polyacrylamide gel and were transferred to polyvinylidene fluoride membranes. The membranes were blocked with 5% skim milk in Tris-buffered saline with 0.1% Tween 20 (TBST) for 1 hour. The membranes were incubated with IκBα, IKK, tubulin (Santa Cruz Biotechnology, CA, USA), phospho-IKK, phospho-IκBα, phospho-JNK, JNK, phospho-ERK1/2, ERK1/2, phospho-p38, and p38 diluted in 5% skim milk in TBST overnight at 4°C. The blots were washed with TBST and incubated for 1 hour with anti-rabbit horseradish peroxidase-conjugated antibodies. Immunoreactive bands were visualized by chemiluminescence using ECL (GE Healthcare, Little Chalfont, Buckinghamshire, UK), according to the manufacturer’s instructions.

### Quantitation of total polyphenols

Total polyphenols from CWE and the size-based fractions were determined by Folin-Ciocalteau (FC) colorimetry as described previously [14]. Gallic acid solutions were used for a calibration standard curve. Twenty microliters of each fraction or total CWE in 1.58 ml of water was incubated with 100 μl of FC reagent (Sigma) for 5 min at room temperature. Three hundred microliters of 1.88 M sodium carbonate solution was used to quench the FC reagent-mediated reaction to form chromogens. After reading absorbance at 765 nm, the concentration of polyphenols was calculated as gallic acid equivalent per gram of extract.

### Statistical analysis

*In vivo* data are presented as mean ± SEM. Statistical differences among the means of multiple groups were determined by using one-way ANOVA followed by the Scheffe test. *In vitro* data are presented as mean ± SD. The difference between the two means was assessed using a non-paired Student’s *t*-test. Calculations were carried out using SPSS version 12. P values of less than 0.05 were considered significant.

## Results

### *In vivo* effect of CWE on levels of serum tumor necrosis factor-α and interleukin-6 upon LPS stimulation

**Figure 1:**
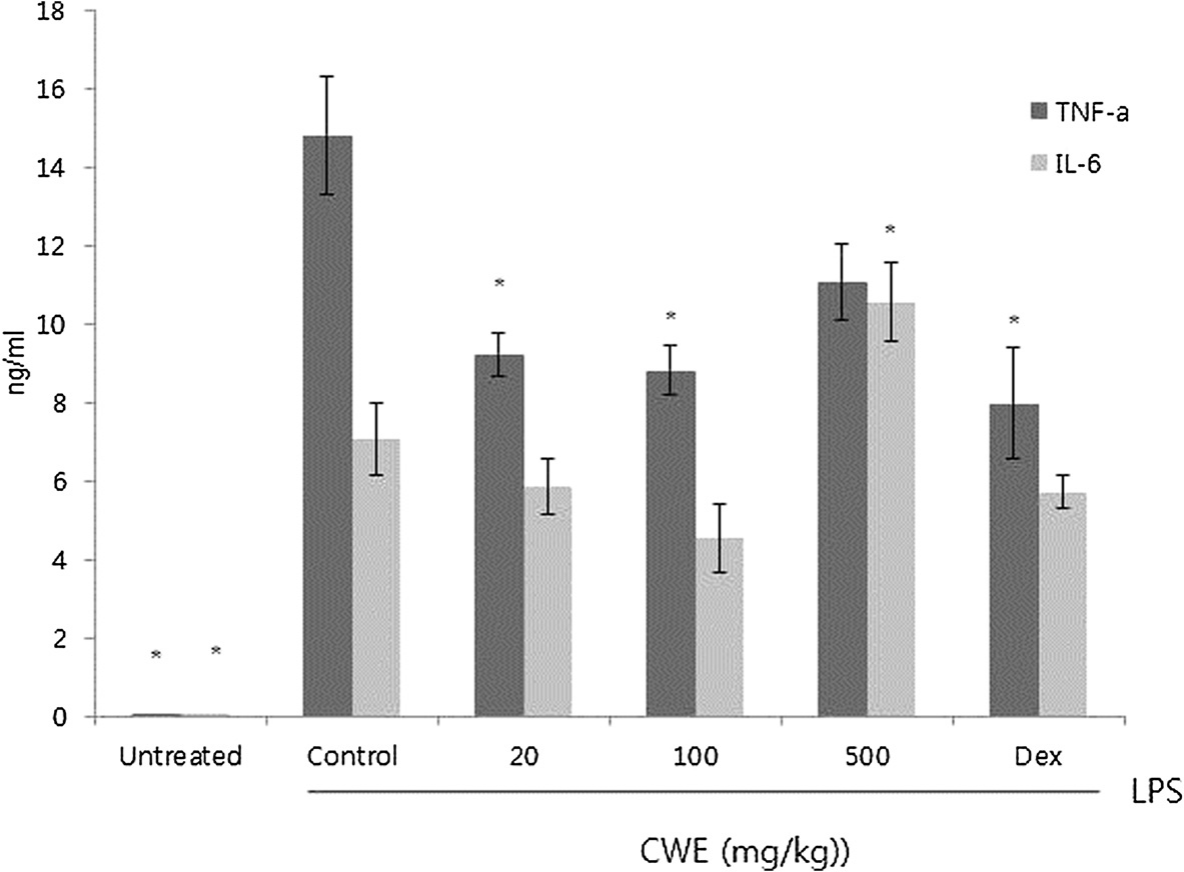
***In vivo *effect of cinnamon water extract(CWE)on levels of LPS**-**stimulated serum inflammatory cytokines**. CWE (20, 100, and 500 mg/kg) was orally administered to mice for 6 days before intraperitoneal injection of LPS (1.3 mg/kg). Dexamethasone (Dex) (5 mg/kg) was i.p. injected 18 h before LPS injection. Serum was obtained 1 h after LPS stimulation and the levels of TNF-α and IL-6 were measured by ELISA. Data are expressed as the mean ± SEM of three independent experiments (n=12). * *P* <0.05 compared with the control group

LPS is an endotoxin originating from the cell walls of gram-negative bacteria, which stimulates the expression of TNF-α and IL-6 in monocytes and macrophages. These cytokines provide protective effects for the body by inducing blood clotting, leukocyte recruitment and activation of adaptive immunity, but a large quantity of such cytokines in serum results in provoking disseminated intravascular coagulation, multiple organ failure or septic shock [15]. First, we tested whether CWE treatment affects systemic inflammatory response to LPS stimulation. To this end, CWE was orally administrated to mice for 6 days before intraperitoneal injection of LPS and the release of serum TNF-α and IL-6 was measured by ELISA. Based on our previous study, doses of 20, 100, and 500 mg/kg were chosen for oral administration [16]. Dexamethasone was used as a reference drug to compare the suppressive activity of CWE in the presence of LPS. Serum levels of TNF-α were significantly reduced with the 20 and 100 mg/kg doses, while a higher dose (500 mg/kg) showed a lesser reduction than the lower dose points and was not statistical significant (Figure 1). This pattern was observed in our previous work in which a dose-dependent reduction in serum IFN-γ occurred only between 20–200 mg/kg of CWE [16]. In the case of IL-6, a dose-dependent decrease occurred in the 20 and 100 mg/kg dosage groups although the latter group reached statistical significance. The 500 mg/kg group showed a higher level of IL-6 than the control group. It seems that unidentified compounds beyond a critical level may interfere with the anti-inflammatory components of CWE.

### *In vitro* effect of CWE on LPS-stimulated TNF-α in peritoneal macrophages

**Figure 2:**
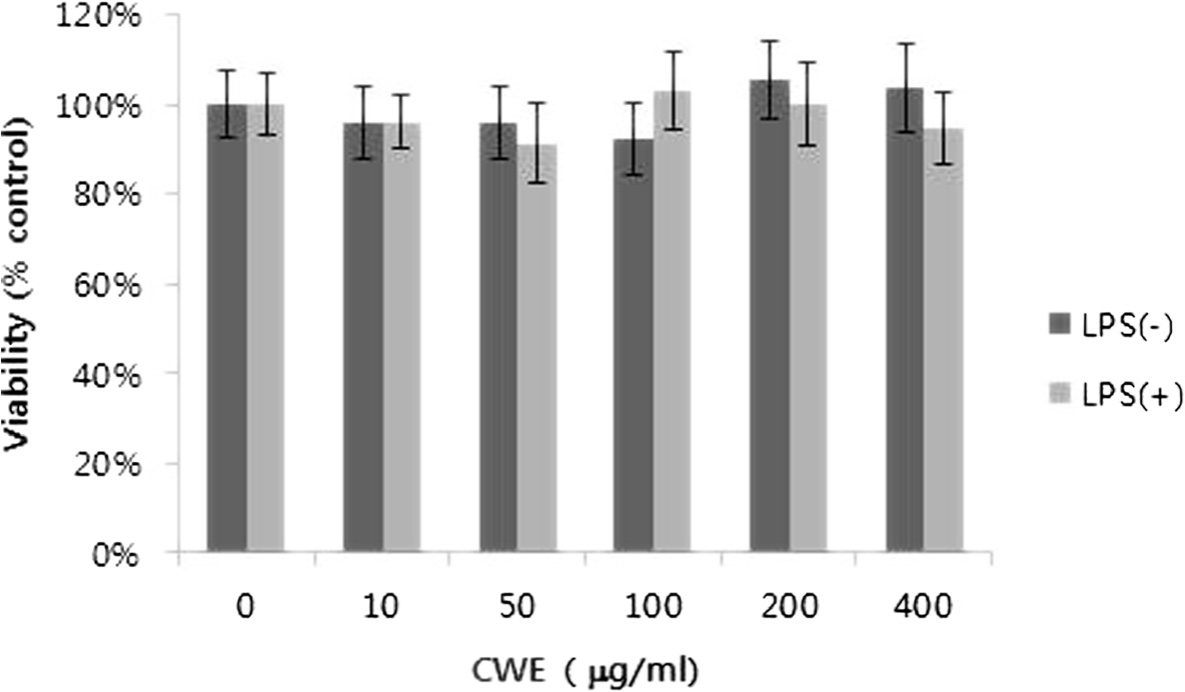
***In vitro *effect of CWE on the viability of macrophages.** Peritoneal macrophages from thioglycollate-injected mice were isolated and treated with varying concentrations of CWE for 24 hours with or without LPS (100 ng/ml). Cell viability was measured by the MTT assay. Control: cells without CWE ( 0 μg/ml). Data are expressed as the mean ± SD of two independent experiments.

**Figure 3:**
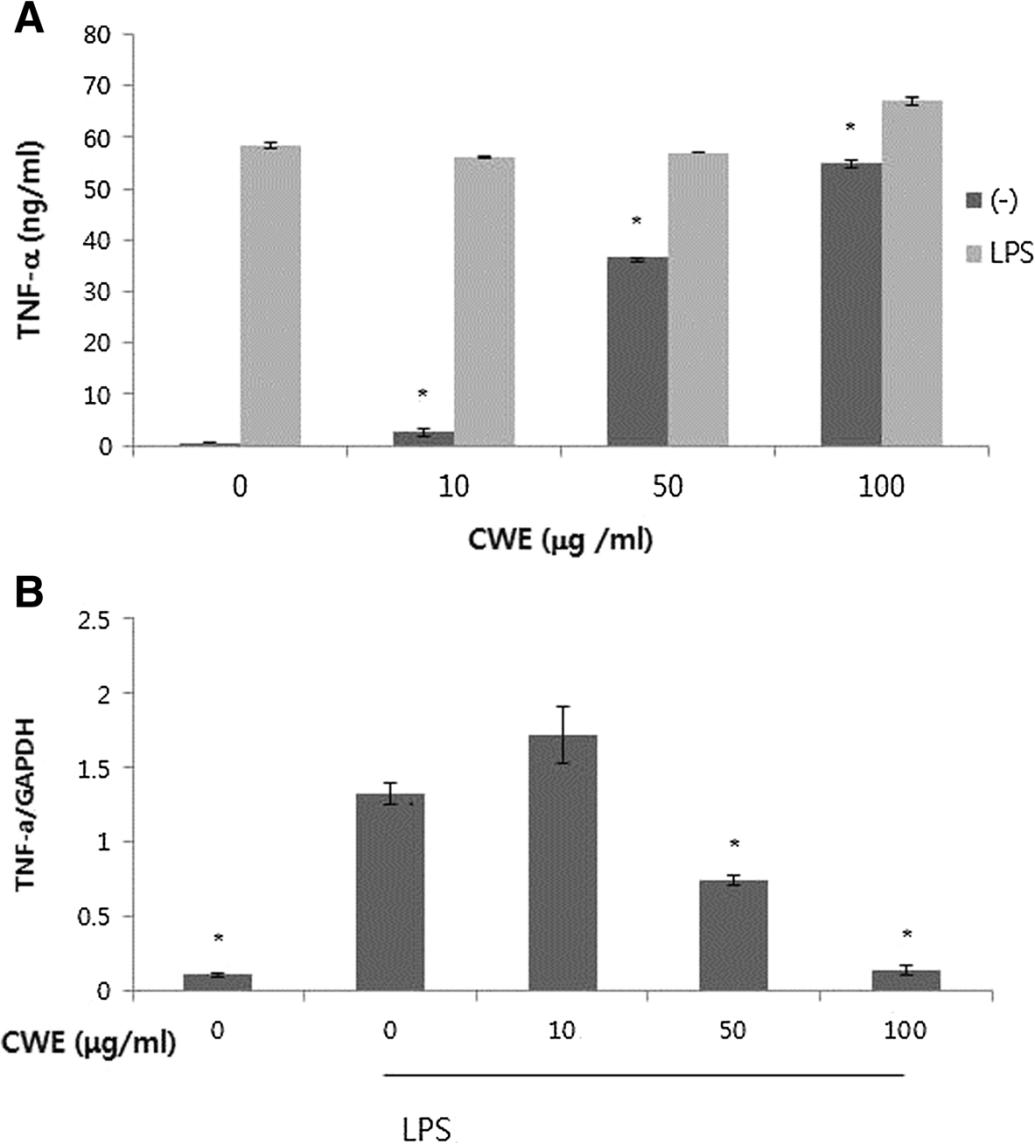
***In vitro *effect of CWE on LPS**-**induced TNF**-**α expression in macrophages.A**: Peritoneal macrophages were treated with CWE for 24 h in the absence or presence of LPS. CWE was added 1 h before LPS stimulation. Supernatants were collected and cytokine levels were measured by ELISA. * *P* <0.05 compared with non-treated cells. **B**: Peritoneal macrophages were pretreated with CWE for 1 h and then stimulated with LPS for 4 h. Total RNA was prepared and realtime RT-PCR was performed. Glyceraldehyde 3-phosphate dehydrogenase (GAPDH) was used as an internal control. * *P* <0.05 compared with cells treated with LPS only. Data are expressed as the mean ± SD of four independent experiments.

Next, we wanted to examine the *in vitro* effect of CWE in macrophages, which are the major cell source of TNF-α. We used peritoneal macrophages isolated from thioglycollate-injected mice to determine the range of CWE concentration representing no cytotoxic activity using the MTT method. Concentrations of CWE up to 400 μg/ml was not cytotoxic to peritoneal macrophages treated with or without LPS (Figure 2). Many herbal water extracts contain polysaccharides, which induce the secretion of TNF-α in non-stimulated macrophages *in vitro*[17]. We found that treatment with CWE alone ranging from 10 to 100 μg/ml stimulated the release of TNF-α into media in a concentration-dependent manner (Figure 3A). Such phenomenon must be due to the presence of water soluble polysaccharides in CWE. Subsequently, we examined the effects of CWE on LPS-stimulated TNF-α secretion. There was no apparent decrease in TNF-α secretion by CWE (Figure 3A). However, TNF-α mRNA expression at 4 h after LPS challenge was decreased in cells treated with 50 and 100 μg/ml of CWE (Figure 3B). Despite its inhibition of LPS-induced TNF-α transcription, the unabated TNF-α levels in supernatant must have been caused by prior exposure of cells to CWE.

### *In vitro* effect of CWE on activation of IκBα, JNK, p38 and ERK 1/2 upon LPS stimulation

**Figure 4:**
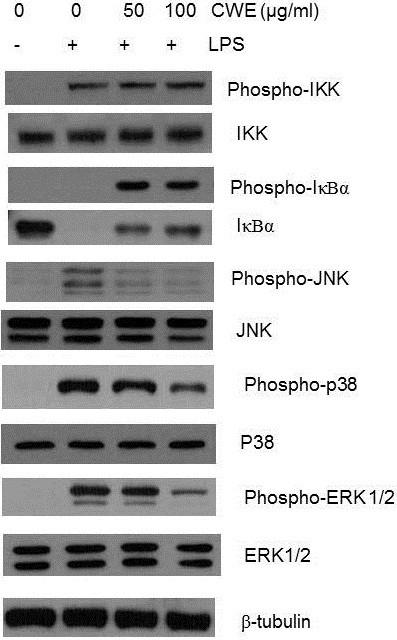
***In vitro *effect of CWE on LPS-induced IκBα degradation and MAP kinase activation.** Peritoneal macrophages were pretreated with the indicated concentration of CWE for 1 h and then stimulated with LPS for 15 min. Phospho-IKK, IκBα, phospho-IκBα,phospho-JNK, JNK, phospho-p38, p38, phospho-ERK1/2, and ERK1/2 in whole protein extracts were examined by Western blot analysis. Tubulin was used as an internal control. One of the five experiments is shown.

In the absence of stimuli, IκBα acts as an inhibitor to block the nuclear translocation of NFκB by masking its nuclear localization signal. Upon inflammatory stimulation, IκBα is subjected to phosphorylation mediated by an upstream kinase, IκBα kinase (IKK), and is subsequently released from NF-κB, followed by phosphorylation-induced proteosomal degradation[18]. We tested the effect of CWE on IκBα degradation 15 min after LPS stimulation. A time course study of IκBα activity in LPS-stimulated macrophages shows that phosphorylation of IκBα reaches its peak at 5 min after LPS stimulation and then disappears at 15 min while a complete degradation of IκBα occurs within 15 min [19]. As expected, an almost complete loss of phospho- IκBα as well as IκBα was identified in macrophages treated with LPS alone (Figure 4). CWE at doses of 50 and 100 μg/ml inhibited IκBα degradation, although phospho-IκBα was still detected. Phosphorylation of IKK was not affected. These results strongly indicate that the inhibitory effect of CWE on NF-κB signaling may occur downstream of the phosphorylation of IκBα.

Mitogen-activated protein (MAP) kinases resident in the cytoplasm of mammalian cells relay extracellular signals to the nucleus through various signal transduction pathways[20]. JNK, p38, and ERK1/2, representing the family of MAP kinases, play a critical role in LPS-induced cytokine gene expression. We tested whether CWE can inhibit the activation of JNK, p38, and ERK1/2 15 min after LPS stimulation. JNK phosphorylation was reduced at all concentrations tested and phosphorylation of p38 and ERK 1/2 was inhibited at 100 μg/ml of CWE, implying that different constituents of CWE may act on these pathways (Figure 4). Taken together, these results show that such inhibition occurred upstream of the MAP kinases.

### Inhibitory effect of the CWE fraction containing high-molecular-weight compounds on signaling molecules in LPS-stimulated macrophages

**Figure 5:**
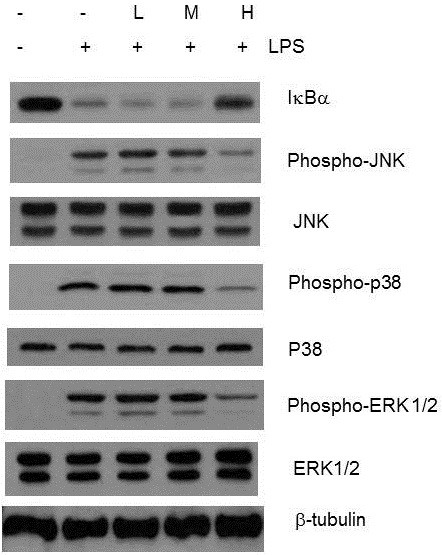
**Effect of size**-**based CWE fractions on signaling molecules.** Peritoneal macrophages were 1 h pretreated with 120 μg/ml of low molecular weight(MW) fraction(L), 30 μg/ml of middle MW fraction (M), and 50 μg/ml of high MW fraction (H) according to the yield ratio relative to total CWE. After LPS stimulation for 15 min, signaling molecules in whole protein extracts were examined by Western blot analysis. One of the five experiments is shown.

Since the effective oral dose of CWE is relatively low, it is reasonable to speculate that the nature of the active components might be small-molecule compounds. Thus we separated CWE into a low MW fraction (below 3 kDa), a middle MW fraction (between 3 kDa and 10 kDa), and a high MW fraction (over 10 kDa). Since the insulin-like and antioxidant activities of CWE originate from polyphenolic compounds, we wanted to determine the polyphenol content in these fractions [12]. As shown in Table 1, the highest concentration of total polyphenols was observed in the high MW fraction, being more than ten-fold than that of the low or middle MW fraction. Next, based on the fraction yield ratio relative to the total CWE, 120 μg/ml, 30 μg/ml and 50 μg/ml of the low, middle and high MW fractions were added to macrophages and their effects on signaling molecules were examined. Suppression of the degradation of IκBα and phosphorylation of JNK, p38 and ERK 1/2 was detected only in cells treated with the high MW fraction (Figure 5). These findings indicate that the inhibitory activity of CWE *in vitro* is derived from the polyphenol-rich fraction.

**Table 1:** **Total polyphenols in CWE and its size**-**based fractions**

**Polyphenols**	**CWE**	**CWE fractions**		
		**Low fraction**	**Middle fraction**	**High fraction**
		(< 3 kDa)	(3–10 kDa)	(> 10 kDa)
(mg/g extract)	88.00 ± 0.58	19.70 ± 0.33	30.59 ± 3.56	341.18 ± 3.33

Total polyphenolic content was measured by Folin-Ciocalteu colorimetry. Results are expressed as means ± SD, n=3.

## Discussion

In this study, we present *in vivo* and *in vitro* evidence that CWE inhibits expression of TNF-α. In addition, LPS-induced IκBα degradation and MAP kinase phosphorylation in macrophages was strongly inhibited by the polyphenol-rich CWE fraction.

Macrophages are phagocytic cells that play a critical role in clearing foreign materials, invading bacteria and cellular debris produced by tissue injuries [21]. Phagocytes such as macrophages contain a variety of pattern-recognition receptors (PRRs), which specifically recognize foreign organisms and modified self ligand. Toll-like receptors (TLRs), complement receptor 3 and scavenger receptors are affiliated members of the PRR family. Among them, LPS uses TLR-mediated signaling pathways such as NF-κB and MAP kinases to stimulate TNF-α and IL-6 in macrophages. Oral administration of CWE decreased serum levels of LPS-induced TNF-α and IL-6, but such anti-inflammatory activity was attenuated in the high dose group. In the clinical setting, CWE is used in combination with other herbal agents and thus different results could be produced. However, our experimental data imply that when used singularly the anti-inflammatory activity of CWE is subjected to dose ranges.

Chronic inflammatory responses found in most autoimmune diseases and metabolic diseases exhibit common characteristic processes where macrophages are initially activated and interferon (IFN)-γ-producing type-1 T helper cells subsequently stimulate macrophages to release more inflammatory cytokines. Together with our previous findings that CWE prevented anti-CD3-stimulated T cells from secreting IFN-γ, our current study clearly shows that CWE is able to interfere with the chronic activation of macrophages [16].

The inhibitory effect of CWE on the signaling pathways mediated by NF-κB and MAP kinases occurred in its polyphenol-rich high MW fraction. There is increasing evidence that polyphenols exert anti-inflammatory effects. Since CWE is rich in polyphenols such as flavonoids and tannins, the anti-inflammatory effect of CWE may originate partly from polyphenolic compounds. Although the high MW fraction accounts for only 25% of the yield of CWE, its polyphenol content is four times more than that of CWE. The high MW fraction may contain polyphenols conjugated with polysaccharides or tannins. Procyanidins, known as condensed tannins, consist of oligomer or polymers of (epi)catechin. A higher degree of polymerized procyanidins exhibited stronger inhibition of macrophage activity [22]. Therefore, it is conceivable that the polyphenol-rich high MW fraction of CWE may contain the anti-inflammatory compounds that play a major role in suppressing LPS-induced NFκB and MAP kinase signaling pathways. Further study is required to examine whether the macromolecular polyphenols of CWE exert these anti-inflammatory effects in animal models.

## Conclusions

In summary, oral treatment of CWE decreased LPS-induced TNF-α and IL-6 release in serum. CWE inhibited IκBα degradation and MAP kinase activation in LPS-stimulated macrophages *in vitro*. In particular, the inhibitory activity of CWE *in vitro* occurred in the polyphenol-rich high molecular weight fraction.

## Abbreviations

CWE: Cinnamon water extract; LPS: Lipopolysaccharide; TNF-α: Tumor necrosis factor- α; IL: Interleukin; DMSO: Dimethyl sulfoxide; ELISA: Enzyme-linked immunosorbent assay; IKK: IκBα kinase; MAP: Mitogen-activated protein; MW: Molecular weight; PRR: Pattern recognition receptor; TLR: Toll-like receptor; IFN: Interferon.

## Competing interests

The authors have no conflict of interests.

## Authors’ contributions

JWH and HK have written the paper. HK and GAY performed in vitro experiments. YBK performed animal experiments. SHE assisted in the preparation of CWE. JHL assisted in the preparation of manuscript. All authors have read and approved the final manuscript.

## Pre-publication history

The pre-publication history for this paper can be accessed here:

http://www.biomedcentral.com/1472-6882/12/237/prepub
